# Association of +331G/A PgR Polymorphism with Susceptibility to Female Reproductive Cancer: Evidence from a Meta-Analysis

**DOI:** 10.1371/journal.pone.0053308

**Published:** 2013-01-22

**Authors:** Sanjib Chaudhary, Aditya K. Panda, Dipti Ranjan Mishra, Sandip K. Mishra

**Affiliations:** 1 Cancer Biology Lab, Department of Gene Function and Regulation, Institute of Life Sciences, Bhubaneswar, Odisha, India; 2 Department of Infectious Disease Biology, Institute of Life Sciences, Bhubaneswar, Odisha, India; Ohio State University Medical Center, United States of America

## Abstract

The progesterone receptor (PgR), a sex steroid hormone receptor that binds progesterone is critical for normal breast development. The PgR (+331G/A, rs10895068) promoter polymorphism is associated with cancer risk possibly by altering the expression of progesterone receptor B isoform. Previous studies have provided inconsistent results. To validate the association between the PgR +331G/A polymorphism and female reproductive cancer risk (breast, endometrial and ovarian cancer), we performed a meta-analysis of 19 studies (19,978 cases and 24,525 controls) by using the CMA Version 2 software. Odds ratios (ORs) and 95% confidence intervals (CIs) were used to assess the strength of the associations. The overall results indicated that the variant allele and genotypes were associated with a mild increase in overall female reproductive cancer risk (A vs. G: OR = 1.063, 95% CI = 1.001–1.129; AA+AG vs. GG: OR = 1.067, 95% CI = 1.002–1.136). The results suggest that the PgR +331G/A polymorphism might be associated with an increased female reproductive cancer risk.

## Introduction

It is now well established that cancer is a multifactorial disease with an orchestrated relationship between genetic and environmental factors [1], [2]. Despite intense study, the number of cancer cases continues to dramatically increase globally. According to American Cancer Society in 2012, more than 500,000 Americans are expected to die from cancer with more than 1500 deaths per day [3].

The progesterone receptor (PgR) is a member of a large ligand-activated nuclear receptor family characterised by a central DNA binding domain, a carboxyl-terminal ligand binding domain and multiple activation (AF) and inhibitory (IF) functional elements. The PgR, which is located on chromosome 11q22, and the oestrogen receptor (ER) have diverse reproductive functions associated with the establishment and maintenance of pregnancy, alveolar development and mammary epithelial development [4], [5], [6]. Progesterone, a steroid hormone, binds to the PgR, causing dimerization and thereby activating its target genes to transcribe through its response elements (PRE). The binding and activation are responsible for the mammary epithelial proliferation [7]. The PgR gene is transcribed by two alternative promoters that translate into two isoforms, progesterone receptor A (PgRA) and progesterone receptor B (PgRB) through which the PgR mediates its physiological functions [6]. The ratio of the expression of the two isoforms is important for the normal physiological function of progesterone signalling, which gets abrogated in cancer [8]. Both isoforms are identical except for the absence of 164 amino acids in N-terminal end of PgRA, which are present in the PgRB. This makes PgRB unique from PgRA because of the presence of AF-3 apart from AF-1 and AF-2, which are normally present in PgRA [9]. Six variable sites, four polymorphism and five common haplotypes have been detected in the PgR gene [10]. The most widely studied polymorphism in the promoter region of the PgR gene is the G to A substitution at position +331 (progesterone receptor, +331 G/A, rs10895068, +331 from ATG start codon). Interestingly, a unique GATA-5 binding site that is adjacent to the +331G/A PgR polymorphism has been found that leads to increased transcriptional activity of PgRB isoforms. *In vitro* over-expression of GATA-5 with hPgR luciferase construct showed greater transcriptional activity with +331AA than with +331GG included in the luciferase construct thereby contributing to breast tumorigenesis [11].

Recently, many studies have investigated the role of the PgR +331G/A polymorphism in the etiology of various types of cancer, i.e., breast, ovary and endometrial cancer. However, the results of these studies remain inconclusive. To clarify the role of +331G/A PgR polymorphism in female reproductive cancers, we performed a meta-analysis of all of the eligible case-control studies to derive overall cancer risk associated this polymorphism (Figure S1).

## Materials and Methods

### Identification and eligibility of relevant study

Recent publications were identified through a literature search using the keywords “Progesterone receptor polymorphism,” “PgR polymorphism” or “PR polymorphism”, and “+331G>A”, or “+331G/A” and “cancer” in Pubmed and cross-references were checked that were not available in Pubmed or Science Direct (published before July 2012). Only those articles written in English were selected for the study. The following criteria were used for inclusion of identified articles for our meta-analysis: (a) evaluation of +331 G/A and cancer risk, (b) case-control study or cohort design and (c) studies that contained available genotype frequencies. The major reason of exclusion of some investigations was the absence of usable data reported in those studies. Finally, the data were extracted from 19 case-control studies totalling to 19,978 cases and 24,525 controls for +331G/A PgR polymorphism.

### Data extraction

Two investigators (SC and DRM) independently extracted all of the data and jointly reached the consensus for all of the articles that were included in the study. Disagreement between the authors was resolved after discussion amongst the authors. The following information was sought from each article: first author's name, year of publication, source of control, country, ethnicity and numbers of cases and controls, genotype frequencies and allele frequencies for each case and control.

### Meta-analysis

The statistical analysis for the current meta-analysis study was performed by using the comprehensive meta-analysis (CMA) V2 software (Biostat, USA). Meta-analysis is a powerful tool which combines results of independent similar studies and derive a definitive conclusion [12]. CMA V2 has several advantages over other software available for computing meta-analyses (http://www.meta-analysis.com/pages/comparisons.html). The strength of the association between the PgR +331G/A polymorphism and risk of cancer was measured by odd ratios (ORs) with 95% confidence intervals (CIs). We examined the association between the allele A of the PgR +331G/A polymorphism and cancer risk, and made comparisons with the dominant genetic model (AA+AG vs. GG). A recessive genetic model was not performed as the data were not available for the homozygous mutant (AA) in the studied cases. The heterogeneity assumption was evaluated with an I^2^-based Cochran's Q statistic test. A significant p value (<0.10) was used to indicate heterogeneity among studies, and the combined OR was calculated by using a random effect model [13]. In contrast, a fixed effect model [14] was used for the calculation of the combined OR for homogeneity among the studies. In addition, the I^2^ statistic was used to quantify inter-study variability. This statistic ranged from 0 to 100%, where a value of 0% indicated no observed heterogeneity, and as the values increased the degree of heterogeneity increased (cut-off points include: I^2^ = 0–25%, no heterogeneity; I^2^ = 25–50%, moderate heterogeneity; I^2^ = 50–75%, large heterogeneity; I^2^ = 75–100%, extreme heterogeneity) [15]. The funnel plot was employed to examine the publication bias. Egger's regression analysis was used for re-evaluation of publication bias, and a P value less than 0.10 was considered to be significant. Funnel plots and Egger's linear regression tests were used to provide a diagnosis of the potential publication bias.

## Results

### Study characteristics

For the association between PgR +331G/A polymorphism and cancer susceptibility, articles were retrieved based on the established search criteria. Nineteen studies met the inclusion criteria, the characteristics of which are reported in Table 1. Of these 19 studies, 10 were breast cancer (BC) studies in which all of the patients were of European ethnicity [11], [16]–[24]; six were ovarian cancer (OC) studies that only included patients of European origin [10], [17], [20], [25], [26], [27] and three studies were endometrial cancer (EC) studies, which enrolled patients of different ethnic groups [28], [29], [30].

**Table 1:** Characteristics of all studies included in meta-analysis.

ID	First author	Year	Country	Ethinic group	Cancer type	Case	Control	HWE		Genotype distribution					Allele distribution (%)			
										Cases			Control		Case		Control	
									GG	GA	AA	GG	GA	AA	G	A	G	A
1	Diegaarde [22]	2008	US	European	Breast Cancer	324	651	NA	294	29		580	70		95.2	4.8	94.4	5.6
2	Feigelson [16]	2004	US	European	Breast Cancer	479	494	0.804	425	53	1	445	48	1	94.2	5.8	94.9	5.1
3	Fernandez [18]	2006	Spain	European	Breast Cancer	544	553	0.927	508	36	0	509	43	1	96.6	3.4	95.9	4.1
4	Huggins [11]	2006	US	European	Breast Cancer	1298	1728	NA	1134	164		1560	168		93.6	6.4	95.1	4.9
5	Johnatty [21]	2008	Australia	European	Breast Cancer	1443	530	NA	1282	161		474	56		94.4	5.6	94.7	5.3
6	Kotsopoulos [23]	2009	US	European	Breast Cancer	1664	2391	<0.001	1463	195	6	2174	202	15	93.7	6.3	95.1	4.9
7	Pearce [17]	2005	US	European	Breast Cancer	1674	2432	0.609	1596	76	2	2317	113		97.6	2.4	97.5	2.5
8	Pearce [17]	2005	US	European	Ovarian Cancer	267	396	NA	243	22	2	353	40	3	95.1	4.9	94.1	5.9
9	Pooley [19]	2006	UK	European	Breast Cancer	4478	4548	0.431	3960	506	12	4005	529	14	94.0	6	93.8	6.2
10	Reding [24]	2009	US	European	Breast Cancer	1264	1021	NA	1128	136		910	111		94.6	5.4	94.5	5.5
11	Romano [20]	2006	Netherlands	European	Breast Cancer	535	379	0.087	476	48	11	339	37	3	93.4	6.6	94.3	5.7
12	Romano [20]	2006	Netherlands	European	Ovarian Cancer	52	379	NA	43	9	0	339	37	3	91.3	8.7	94.3	5.7
13	Ludwig [27]	2009	Poland	European	Ovarian Cancer	215	352	>0.05	183	32		312	39	1	92.5	7.5	94.1	5.9
14	Terry [10]	2004	US	European	Ovarian Cancer	920	960	NA	831	87	2	868	91	1	95.0	5	95.1	4.9
15	Risch [26]	2006	US	European	Ovarian Cancer	490	534	NA	426	61		489	44		93.1	6.9	95.6	4.4
16	Berchuck [25]	2004	US	European	Ovarian Cancer	438	504	0.53	400	37	1	445	58	1	95.5	4.5	94.0	6
17	Berchuck [25]	2004	Australia	European	Ovarian Cancer	535	298	0.27	483	48	4	266	30	2	94.7	5.3	94.2	5.8
18	O'Mara [29]	2010	Australia	European	Endomertrial cancer	2757§	4642§	NA	1058	148	7	1185	160	3	93.3	6.7	93.8	6.2
19	O'Mara [29]	2010	US	European	Endomertrial cancer			NA	412	41	2	1567	121	13	95.0	5	95.6	4.4
20	O'Mara [29]	2010	UK	European	Endomertrial cancer			NA	966	119	4	1392	192	9	94.1	5.9	93.4	6.6
21	Vivo [28]	2002	US	European	Endometrial cancer	187	885*	NA	158	28	0	787	98		91.9	8.1	94.4	5.6
22	Lee [30]	2010	US	African American	Endometrial Cancer	578§	1901§	NA	43	0	0	269	8	0	100	0	98.5	1.5
23	Lee [30]	2010	US	Jap. American	Endometrial Cancer				73	0	0	327	0	0	100	0	100	0
24	Lee [30]	2010	US	Native Hawaiins	Endometrial Cancer				15	0	0	187	13	0	100	0	96.7	3.3
25	Lee [30]	2010	US	Latinos	Endometrial Cancer				61	4	1	260	18	1	95.4	4.6	96.4	3.6
26	Lee [30]	2010	US	Whites	Endometrial Cancer				60	9	1	269	26	0	92.1	7.9	95.5	4.5
27	Lee [30]	2010	US	CTS Whites	Endometrial Cancer				268	42	1	457	64	2	92.9	7.1	93.4	6.6

* 506 women who were controls in a nested case-control study of breast Cancer were also included along with 397 controls.

§ Represents total number of cases and control in a study.

### Publication bias

Begg's Funnel plot and Egger's test was conducted to assess whether there was any publication bias in the studies included in our meta-analysis. The shape of the funnel did not elucidate any obvious asymmetry in all of the comparison models. Thereafter, Egger's test was used to provide statistical evidence of the funnel plot symmetry and did not show any publication bias (Table 2).

**Table 2:** Statistics to test publication bias and heterogeneity in meta-analysis.

SNP	Study	Sample Size			Egger's regression Analysis			Heterogeneity analysis		
		Case	Control	intercept	95%CI	p value	Q value	P*h*	I2 (%)	Model
A vs. G	Overall	19978	24525	0.18	−0.74 to 1.12	0.68	31.08	0.186	19.58	Fixed
AA+AG vs. GG	Overall	19978	24525	0.1	−0.86 to 1.08	0.81	33.67	0.18	25.76	Fixed

### Heterogeneity test

Q-test and I^2^ statistics were used to test for heterogeneity among the studies. No heterogeneity was observed in either allele (A vs. G) as well as the dominant genotype model for overall cancer, which was included for the analysis (Overall allele, A vs. G: Q = 31.08, P_heterogeneity_ = 0.186, I^2^ = 19.58; Overall dominant model, AA+GG vs. GG: Q = 25.76, P_heterogeneity_ = 0.11, I^2^ = 25.76).

### Meta-analysis result

**Figure 1:**
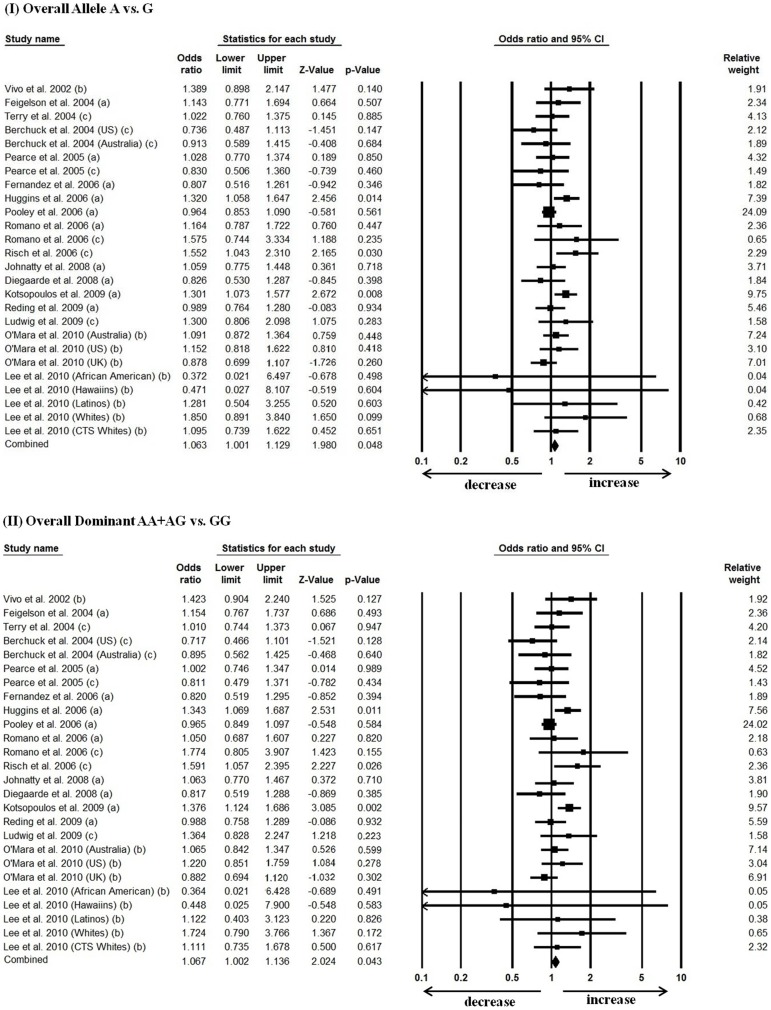
Forest plot of overall cancer risk associated with +331G/A PgR polymorphism. The squares and horizontal lines correspond to the study-specific OR and 95% CI. The area of the squares reflects the weight of the respective study. The diamond represents the pooled OR and 95% CI. Forest plots evaluating the association of overall allele (I), and dominant (II) genetic model with cancer risk are presented. Breast cancer, endometrial cancer and ovarian cancer denoted as a, b, and c respectively.

Our meta-analysis identified an evident mild association between PgR (+331G/A) polymorphism and an increased cancer risk. Compared with the wild-type G allele, the overall variant A allele was associated with a mild increased cancer risk (OR = 1.063, 95% CI = 1.001–1.129, p = 0.048) [Figure 1 (I)]. In addition, the dominant model (AA+AG vs. GG) too showed a modest association between the polymorphism and an increased cancer risk (OR = 1.067, 95% CI = 1.002–1.136, p = 0.043) [Figure 1 (II)].

## Discussion

Genetic epidemiological studies have suggested the relationship between different SNPs and disease. However, robust statistical powers with genotype-phenotype investigation are essential to detect the mild, moderate or strong association with the disease. The progesterone hormone mediates its physiological function through the PgR, a steroid receptor of class I nuclear receptor super-family. Two different isoforms of PgR (PgRA and PgRB), which are transcribed from two different promoters of the same gene [31], [32] are co-expressed in equal levels in normal mammary epithelial [33], ovarian [34] and endometrial cells [35]. The relative expression of this two isoform is altered in malignant transformation. The PgR +331 G/A polymorphism in the promoter results in the introduction of a TATA box that selectively enhances the production of the PgRB isoform, causing an altered PgRA: PgRB protein ratio [36]. The PgR +331 G/A promoter polymorphism has been extensively studied because of its relationship in different cancers such as breast, endometrial and ovarian cancer. However, the results from these studies were ambiguous. Apart from the PgR +331 G/A polymorphism, there are additional polymorphisms such as +44C/T, S344T, G393G, V600L, H770H and Alulns allele [37]. We evaluated the +331G/A PgR promoter polymorphism for two reasons: (1) its association with many cancers like endometrial, ovarian and breast cancer, although there are conflicting observations and (2) the functional attribute of +331G/A PgR has been delineated and has been found to play an important role in hormone related cancer.

In previous published pooled studies, Yang and colleagues have reported an association between PgR +331 G/A variant and breast cancer risk [38]. However, the other meta-analysis reviewed an additional 4 studies and did not find this association [39]. On the basis of availability of more studies on PgR +331 G/A polymorphism and female hormone dependent cancer, we did a meta-analysis to determine conclusively whether an association exists between them. We detected a mild association between PgR +331 G/A polymorphism with overall female cancer risk. As all the studies involved women of only Caucasian origin, we did not perform any subgroup analysis by ethnicity. The prevalence of a minor allele A is restricted to only the European population (5–10%) and is rare in women of African, Asian and Middle Eastern descent which might be the reason for mild association even if the included studies are high. [40]. The non-detection of association between the PgR +331 G/A polymorphism and breast cancer was consistent with the previous study [39].

A genome-wide association studies (GWAS) have been carried out for various cancers (breast, prostate, colorectal, and lung) and several susceptible alleles have been identified [41]. None of the GWAS have identified the +331 G/A PgR polymorphism as a risk factor for cancer predisposition despite this functional SNP has been established. In contrast, our meta-analysis results revealed an association of the PgR (+331 G/A) polymorphism with a susceptibility to cancer. Corroborating with current study, discrepancies between the GWAS and candidate gene association studies (CG) have been reported for different disease [42], [43], [44], [45]. CG studies tend to have higher statistical power than the power in GWAS [46].

The progesterone hormone exerts its physiologic effect exclusively through the presence of the PgR as shown in mouse models [47], [48]. The PgR isoforms, PgRA and PgRB, display different transactivation properties based on the cell type and target promoter [49]. PgRB functions as a strong transcriptional activator of the PgR-dependent promoters in PgRA-inactive cell types, but the agonist-bound PgRA under this condition can repress the PgRB transcriptional activity and other steroid receptors like ER α [50]. Progesterone acts as an antagonist of oestrogen (E2)-induced cell proliferation of uterine epithelium through PgRA [48]. Treating PRAKO^−/−^ mice (deficient in PgRA) with E2 and progesterone resulted in a progesterone-dependent cell proliferation, which was not detected in E2 and progesterone–treated wild-type or PRAKO mice, suggesting that PgRB has a role in uterine endometrium proliferation. Furthermore, the PgRB isoform could also elicit normal proliferation and differentiation as observed in PRAKO^−/−^ mammary epithelium through progesterone. During endometriosis, only the PgRA isoform is expressed and the absence of PgRB is suggested due to the inappropriate cycling of the endometrial gland [25]; however PgRA expression in normal cycling is predominant during the proliferative phase, which shifts towards PgRB expression during the early secretary phase [33]. The upregulation and proliferative nature of the PgRB isoform in the carriers of the +331A allele may result in different types of cancer [25]. With respect to this biological postulation, the +331G/A PgR may modulate cancer risk. However, the association of this polymorphism with overall cancer risk was mild (OR = 1.063, p = 0.048). It is important to note that expression of the PgR is associated with better disease-free survival [51]. An alteration in the ratio of the expression of the PgR isoforms precedes changes that may lead to endometrial carcinoma [52]. The increase in PgRB expression due to polymorphism in promoter leads to changes in the isoform ratio and is associated with an increased risk of developing endometrial cancer [53]. Additionally, a case-control study of endometrial cancer with the +331G/A PgR polymorphism and PgR expression predicted that the recurrence risk and clinical response to progesterone therapy was six times more likely in women with PR(+) than with PR(−) tumors. Progesterone therapy is effective against the developed endometrial cancer, which is dependent on oestrogen-mediated proliferation. Also, PR(+) endometrial cancer was an independent prognostic factor in disease-free survival [54]. The finding that PgRB could contribute to cell proliferation may provide clinical implications for hormonal management of endometrial and mammalian epithelium by rectifying relative expression of the PgRA: PgRB isoform, which is essential for appropriate reproductive tissue responses. Isoform-specific modulators like progestin, which could differentiate between PgRA and PgRB isoform, may also be of clinical value.

Although earlier reports on the association of PgR (+331G/A) polymorphism and cancer are inconsistent, the current meta-analysis has provided a definitive conclusion. Inconsistencies among the CG association studies could be attributed to small sample sizes, improper clinical categorisation and the inclusion of different ethnicities.

### Conclusion

A mild association between PgR +331G/A polymorphism and female reproductive cancer risk was detected. As the eligible case-control studies cannot provide a casual relationship, large and well-designed genotype-phenotype studies on different cancer are necessary to derive a definitive role of this SNP with cancer.

## Supporting Information

Checklist S1 **PRISMA 2009 checklist.**
(DOC)
